# Cardiovascular Disease as a Consequence or a Cause of Cancer: Potential Role of Extracellular Vesicles

**DOI:** 10.3390/biom13020321

**Published:** 2023-02-08

**Authors:** Elisabeta Badila, Cristina Japie, Ana-Maria Vrabie, Adrian Badila, Adriana Georgescu

**Affiliations:** 1 “Carol Davila” University of Medicine and Pharmacy of Bucharest, 050474 Bucharest, Romania; 2 Cardiology Department, Colentina Clinical Hospital, 020125 Bucharest, Romania; 3 Internal Medicine Department, Bucharest Emergency Clinical Hospital, 014461 Bucharest, Romania; 4 Orthopedic and Traumatology Clinic, Bucharest Emergency University Hospital, 050098 Bucharest, Romania; 5 Pathophysiology and Pharmacology Department, Institute of Cellular Biology and Pathology “Nicolae Simionescu” of the Romanian Academy, 050568 Bucharest, Romania

**Keywords:** cardiovascular disease, cancer, cardio-oncology, extracellular vesicles, microvesicles, exosomes, biomarkers

## Abstract

Both cardiovascular disease and cancer continue to be causes of morbidity and mortality all over the world. Preventing and treating heart disease in patients undergoing cancer treatment remain an important and ongoing challenge for improving the lives of cancer patients, but also for their survival. Despite ongoing efforts to improve patient survival, minimal advances have been made in the early detection of cardiovascular disease in patients suffering from cancer. Understanding the communication between cancer and cardiovascular disease can be based on a deeper knowledge of the molecular mechanisms that define the profile of the bilateral network and establish disease-specific biomarkers and therapeutic targets. The role of exosomes, microvesicles, and apoptotic bodies, together defined as extracellular vesicles (EVs), in cross talk between cardiovascular disease and cancer is in an incipient form of research. Here, we will discuss the preclinical evidence on the bilateral connection between cancer and cardiovascular disease (especially early cardiac changes) through some specific mediators such as EVs. Investigating EV-based biomarkers and therapies may uncover the responsible mechanisms, detect the early stages of cardiovascular damage and elucidate novel therapeutic approaches. The ultimate goal is to reduce the burden of cardiovascular diseases by improving the standard of care in oncological patients treated with anticancer drugs or radiotherapy.

## 1. Introduction

Cardiovascular (CV) diseases and cancer are two of the most common causes of death worldwide. The World Health Organisation estimates that 17.9 million deaths per year are attributed to CV disease and nearly 10 million deaths are caused by cancer [1,2]. However, because of early detection and diagnosis and the discovery of novel therapeutic approaches, the mortality rate for these two types of pathologies, although still high, has reduced in recent years [3].

Cardiovascular disease prevention for cancer patients has become an increasingly important concern in the ongoing struggle to improve patients’ quality of life. This brings together experts in the fields of cardiology and oncology to provide timely and specialized CV care to oncological patients and survivors of all ages. 

By addressing the intersection of the CV and oncologic fields, a comprehensive CV risk assessment for identification of cancer patients who are at high risk for suffering cardiac toxicities from cancer therapies can be pursued. Studies show that in some types of cancer, the mortality cause by other comorbidities, such as CV disease, is higher than the mortality of cancer itself [4]. This can be explained by the development of cardiotoxicity, which translates to the alteration of cardiac structure and function following chemotherapy, radiotherapy or immunotherapy, used either alone or in combination.

The long-term follow-up of cancer survivors who received cardiotoxic treatments must be taken into consideration for early identification and management of CV disease. More specifically, in addition to the use of advanced imaging for early identification of changes in cardiac function during cancer treatment, the identification of new biomarkers could yield important benefits in the survival of cancer patients. Recent studies have shown that the incidence of cancer in patients with CV disease is also increasing [5]. This means that one disease may influence the other, thus, leading to a vicious cycle.

Extracellular vesicles (EVs) could be among the most important biomarkers as they play a decisive role in early identification and determining the CV risk for cancer patients receiving specific treatment for this disease. In addition to their role as mediators in intercellular communication through the transfer of bioactive molecules from secretory cells of origin to recipient cells, modulating multiple pathophysiological processes and serving as biomarkers for doxorubicin (DOX)- induced cardiotoxicity, EVs can serve as both biomarkers for doxorubicin (DOX)-induced cardiotoxicity, and carriers of certain proteins, such as miRNA and lncRNA and certain chemotherapeutic drugs in decreasing the dosage of DOX and alleviating cardiotoxicity. 

This review briefly describes some aspects of CV disease development in patients with cancer and highlights the potential role of EVs as diagnostic biomarkers and drug delivery vehicles. In order to establish the specific role of EVs in the dialogue between CV disease and cancer, fundamental, clinical and translational research has been recently encouraged and consolidated.

## 2. Risk Factors Shared by Cardiovascular Disease and Cancer

As people continue to age, more patients present with two or more coexisting chronic diseases or conditions (frequently referred to as multimorbidity). Hence, the course of one disease may influence the development or the evolution of the other coexisting diseases.

**Figure 1:**
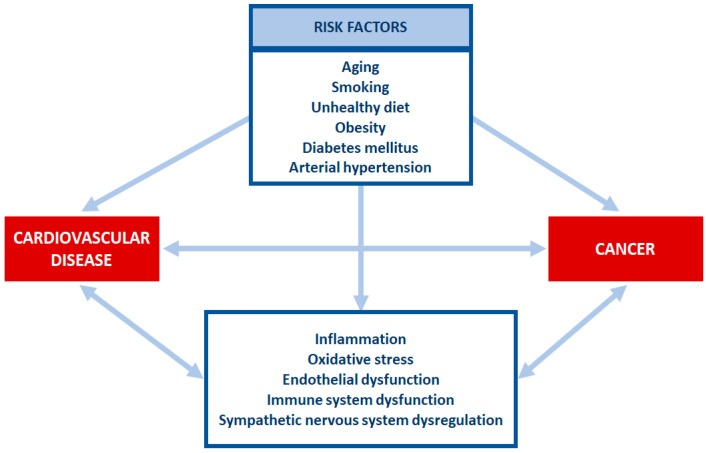
Risk factors and mechanisms shared by cardiovascular disease and cancer.

Many chronic diseases share a series of risk factors. When taken alone, a risk factor may display a small contribution to the development of a certain disease, but the combination of them could increase the risk of multimorbidity [6]. CV disease and cancer share a series of risk factors such as aging, smoking, unhealthy diet, obesity, diabetes mellitus and arterial hypertension (Figure 1). The combination of these risk factors increases the risk of both CV disease and cancer [7].

### 2.1. Aging

Dysfunction of the immune system increases with age and is strongly associated with a poor response of the organism to pathogens and, thus, the development of inflammation, which leads to multimorbidity, such as CV disease, cancer and Alzheimer’s disease [8]. These diseases involve an elevated systemic inflammatory state (with high levels of proinflammatory cytokines and acute phase reactants) and, hence, lead to a vicious cycle [8]. The activation of age-associated chronic inflammation has been named “inflammaging” [8]. In addition to the dysfunction of the immune system, aging also involves dysregulation of the sympathetic nervous system, which may contribute to “inflammaging” [9]. 

### 2.2. Smoking

Smoking is associated with oxidative stress, inflammation, endothelial dysfunction and a prothrombotic status and, thus, leads to CV disease (arterial hypertension, atherosclerosis, coronary heart disease, aortic and peripheral artery disease, stroke) and/or cancer (e.g., lung and bladder cancer) [10]. Nicotine use inhibits apoptosis, stimulates angiogenesis and increases the risk of cancer [11]. It is estimated that 80–90% of lung cancer mortality and 30% of all cancer mortality is caused by smoking, whereas long-term exposure to smoke increases the risk of more than 17 types of cancer [12].

### 2.3. Unhealthy Diet

An unhealthy diet increases the risk of both CV disease as well as cancer. It has been shown that consumption of processed red meat (beef, pork, lamb, veal) is associated with atherosclerosis and linked to the development of colorectal cancer [13]. High sodium intake is associated with increased blood pressure values, but lately studies have reported that a moderate intake of salt can significantly increase the risk of gastric cancer [14]. Alcohol consumption can lead to dilated cardiomyopathy, but is also related to the development of different types of cancer such as esophageal, liver, colorectal, pharyngeal or laryngeal [15].

### 2.4. Obesity

Obesity is a global epidemic and often referred to as “globesity” [16]. The World Health Organization estimates that more than 1 billion people worldwide are obese, with an alarming increasing trend among children (650 million adults, 340 million adolescents and 39 million children) [17]. 

Obesity is a risk factor for many chronic diseases (type 2 diabetes mellitus, arterial hypertension, stroke, various forms of cancer) and, also for premature death. Obesity is a proinflammatory state that implies the activation of inflammatory cytokines such as tumour necrosis factor-alpha (TNF-α), interleukins type 6 and 18 (IL-6 and IL-18, respectively), resistin or visfatin [18]. In addition, interleukin-6 stimulates the production of C-reactive protein [18]. Leptin is a hormone produced primarily by fat tissue and its circulating levels are directly proportional to the amount of energy stored as fat within the human body. When leptin levels increase, it displays proinflammatory action in immune cells such as macrophages and lymphocytes [19]. Furthermore, chronic inflammation can lead to alteration of the deoxyribonucleic acid (DNA) and, thus, increase the risk of mutations and consequently the risk of cancer [20].

It is estimated that 20% of cancers are related to obesity [21]. Adipose tissue can be considered a large endocrine organ that promotes the production of estrogen and, hence, the increased risk of ovarian and breast cancer in postmenopausal women [22]. Studies have shown that obesity is also linked to other forms of cancer: colorectal cancer (especially in men), endometrial, esophageal adenocarcinoma, gall bladder and renal cancer [23].

### 2.5. Diabetes Mellitus

Diabetes mellitus influences CV disease and may increase the risk of cancer. This can be explained through increased inflammation, enhanced oxidative stress, hyper-insulinemia and hyperglycemia [24]. Studies have shown that cancer is more frequent in diabetic patients versus non-diabetic ones and more frequent in those treated with insulin (either in monotherapy or in association with oral antidiabetic drugs) [25]. Serum insulin growth factor (IGF) levels are increased as chronic hyperinsulinemia leads to decreased levels of IGF-binding proteins. Tumour cells express both insulin receptors and IGF receptors [26]. Increased levels of IGF can contribute to the development of various types of cancer by promoting cell proliferation (e.g., colorectal, prostate, esophageal, pancreatic and premenopausal breast cancer) [27]. On the other hand, the use of metformin (an insulin sensitizer), reduces it, whereas use of sulfonylureas (insulin secretagogues) appears to increase the risk for cancer in individuals with type 2 diabetes [28]. The postulated mechanism of the protective effect of metformin on cellular neoplasia was inhibition of the adenosine monophosphate-activated protein kinase/liver kinase B-1 (AMPK/LKB1)-dependent growth pathway [29]. 

### 2.6. Arterial Hypertension

Arterial hypertension is a well-established risk factor for CV disease. The role of angiotensin II (Ang II) in hypertension has been extensively discussed, but studies demonstrate that Ang II is also involved in the production of vascular endothelial growth factor (VEGF) [30]. The latter stimulates angiogenesis and acts as an autocrine growth factor, being overexpressed in around 80% of patients with renal cell carcinoma [31]. Tumourigenesis is also increased as a consequence of increased oxidative stress caused by the hypertensive state. Thus, hypertension may be considered as another possible link between CV disease and cancer [30]. 

A possible association between hypertension and breast cancer has been suggested, with a higher risk of post-menopausal hypertensive women to develop breast cancer. A potential pathophysiological mechanism involved is the increased production of inflammatory mediators through adipose tissue, which is more pronounced in post-menopausal women [32]. A large cohort study on 577,799 patients, with a follow-up of 12 years, demonstrated that each increase of 10 mmHg in blood pressure values in both men and women was associated with cancer-related mortality and with an increased risk of cancer only in men [33]. 

A large number of patients developed new onset hypertension or showed an increase in blood pressure values in periods when chemotherapy drugs (such as VEGF inhibitors, alkylating agents, erythropoietin, immunosuppressive agents, steroids) were administered compared with periods with no chemotherapy. A possible explanation may be that chemotherapy disrupts angiogenesis and acts directly on vascular function [34].

## 3. Cross Talk between Cardiovascular Disease and Cancer

Traditionally, the interest for cardio-oncology developed after the acknowledgement of CV side effects induced by chemotherapy drugs. In this view, CV disease and cancer were depicted as two separate entities and the link between them was unidirectional, represented by the intensely studied field of cancer therapy-related CV toxicity. Moreover, cancer itself may be predisposed to CV disease development through direct mechanisms. However, over the past years, the field of cardio-oncology has significantly evolved, with the consideration that CV disease may not only be a consequence of cancer, but it may itself predispose patients to cancer development. The term “reverse cardio-oncology” has emerged, as we began to understand that the systemic effects of CV disease can have pro-tumourigenic effects [35]. Data from major clinical trials indicated that cancer was the leading cause of non-CV mortality, accounting for more than 35% in both TOPCAT and PARADIGM-HF trials [36,37]. In the RE-LY trial, more than one third of deaths among patients with atrial fibrillation were caused by non-CV causes, with cancer being a leading cause [38]. Epidemiological data have reported an association between CV disease and an increased risk of developing cancer, suggesting that CV disease may enhance an oncogenic process through various pathophysiological pathways, including numerous factors secreted by the heart.

### 3.1. Myocardial Infarction

Combining two or more of the aforementioned risk factors may increase the risk to develop acute cardiac events, such as myocardial infarction (MI). A prospective cohort study included 28,763 patients without a previous history of MI that were followed from baseline to date of cancer, death, migration, or study end, for a mean period of 15.7 years [39]. During follow-up, 1747 patients suffered MI and 146 of them developed cancer. Patients with MI had an increased risk of 46% to develop cancer compared to those without MI. The incidence of cancer was the highest in the first 6 months after MI. Another prospective study that included 1081 patients with MI that were followed for almost 5 years demonstrated that the patients who subsequently developed heart failure (HF) had a higher incidence of cancer compared to those without HF [5]. Both studies stated that patients with a history of MI have a higher risk of cancer even after a significant period of time after MI [5,39]. The clarification comes from a study conducted on APC^min^ mice, which are prone to developing precancerous intestinal tumors, with HF induced by inflicting large anterior MI, where it was shown that myocardial ischemia and necrosis contribute to inflammation and angiogenesis and increase the levels of TNF, thus leading to the development of cancer [40].

Another study that began in 1996 included all Danish residents aged 30–99 (2,871,168 subjects) without prior MI or cancer, and they were followed up for 16 years [41]. Of all subjects, 122,275 suffered MI during follow-up, 11,375 developed subsequent cancer and 65,225 subjects died. In the reference population, 372,397 subjects developed cancer and 753,767 died [41]. MI was associated with an increased risk of cancer after adjusting for age, sex or chronic obstructive pulmonary disease, arterial hypertension, dyslipidemia, diabetes and socio-economic status, but not after further adjustment for the first 6 months after MI [41].

On the contrary to the abovementioned studies showing an increased risk of cancer in patients after MI, various types of cancer treated with antineoplastic pads containing sodium nitroprusside were associated with reduced mortality and improved outcome after MI (12.3% mortality with pads versus 29.9% without pads) [42]. 

### 3.2. Heart Failure

Heart failure is a clinical syndrome characterized by signs (peripheral oedema, pulmonary crackles, jugular venous distention) and symptoms (dyspnea, orthopnea, fatigue) but also by an abnormal structure and/or function of the heart. Patients with both preserved and reduced ejection fraction HF display a poor prognosis with an estimated 50% and 25% survival rate at 5 years from diagnosis and 10 years, respectively [43]. However, it seems that 20% and up to 50% of patients with HF die from non-CV causes [44]. 

A retrospective cohort study in Germany between 2000 and 2018 included 100,124 patients with HF and 100,124 patients without HF [45]. In total, 25.7% of patients with HF and 16.2% of patients without HF were diagnosed with cancer. HF is associated with an increased incidence of all cancer sites, the strongest association being for lip, oral cavity, pharynx and lung cancer [45]. 

Another study that took place between 2002 and 2018 included 103,421 patients with HF and 104,012 control subjects with no cancer within three years before baseline, who were followed for more than five years [46]. A total of 12,036 patients with HF were newly diagnosed with cancer compared to 7045 control subjects. HF patients showed increased incidence of lung, liver and nervous system cancer, with a similar spread in both men and women. Cancer mortality was also higher in HF patients, especially in those younger than 70 years old (a total of 103,608 deaths, 64,483 patients with HF versus 39,125 control subjects) [46]. The cause of death was known in 74.5% of all deaths. In total, 5946 of deaths were caused by cancer (4738 in patients with HF versus 1208 in the control group) and 12 575 deaths were caused by HF (9703 in patients with HF versus 2872 in the control group) [46]. All of these studies show that HF patients have a higher rate of cancer than healthy control populations. Going further with this idea, we could suggest that HF might actually cause cancer. 

However, the vast majority of studies shows that cancer patients develop HF as a consequence of cancer treatment, whereas the studies showing that patients with HF may have an increased incidence of cancer during the course of the disease are quite few. This could be plausible from a biological point of view because both HF and cancer have common risk factors such as obesity and diabetes, but also inadequate lifestyle, including smoking, alcohol consumption and lack of physical activity. Moreover, the factors secreted by the failing heart could stimulate tumor growth. Therefore, because of the high incidence of both diseases and their impact on the lives of those affected, these patients deserve the maximum joint efforts of cardiologists and oncologists.

### 3.3. Cardiovascular Medication and Cancer

As discussed above, various risk factors, such as arterial hypertension, dyslipidemia or diabetes mellitus may promote the development and progression of cancer. However, studies have also shown that certain classes of medication used to treat CV disease may play a role in either promoting or inhibiting the development of cancer.

Antiplatelet drugs, such as aspirin, are used in the primary and secondary prevention of CV disease. As far as cancer is concerned, their use must be done with caution depending on the dose used and the age of the patients. There is rising evidence regarding the use of low-dose aspirin and cancer prevention. Thus, the studies have shown that the use of aspirin for more than five years may be correlated with a reduced risk of colorectal cancer, whereas the longer use (up to 20 years) is associated with even higher protection [47]. The mechanism of action of aspirin in preventing cancer remains to be elucidated. However, we could speculate that long-term administered aspirin could have a positive effect on cells with malignant potential and could suppress certain inflammatory processes. On the other hand, the use of aspirin in patients over 70 years old has been associated with cancer progression and/or mortality because of its risk of bleeding [48]. 

Diuretics, in general, do not influence the risk of cancer, but there is evidence that treatment with hydrochlorothiazide may increase the incidence of skin cancer beucase of its photosensitivity in a dose-dependent manner [49,50]. Thiazide diuretics are associated with insulin resistance, which is considered a risk factor for breast cancer [51]. The use of diuretics for more than ten years may increase the incidence of breast cancer by 16%, although other studies show the opposite, that long-term use of diuretics protects against breast cancer [52,53]. More studies are needed in order to elucidate the mechanism behind these statements and establish whether diuretics play a protective role or not against breast cancer.

Calcium channel blockers (CCBs) are a widely used class of medication for treating CV disease. CCBs inhibit apoptosis and interfere with cell differentiation via calcium triggering signals [54]. Studies evidenced that treatment for more than nine years with CCBs increases the risk of cancer, especially lung and prostate cancer in men and breast cancer in women (particularly invasive ductal carcinoma and invasive lobular carcinoma) [55,56]. However, one recent study showed no association between the use of CCBs and an increased risk of breast cancer compared with non-CCB antihypertensive drugs [57]. 

Selective beta-2 blockers may reduce the recurrence and metastasis of malignancies, improving overall survival in cancer, whereas beta-1 blockers showed no beneficial effect on cancer [58]. Non-selective beta-blockers reduced the incidence of hepatocellular carcinoma in patients with cirrhosis by inhibiting angiogenesis and decreasing bacterial translocation [59]. Both selective and non-selective beta-blockers, particularly propranolol, were associated with a reduction of mortality rate in pancreatic cancer, probably by inhibiting the damage induced by catecholamine stimulation of the adrenoreceptors [60]. Propranolol may reduce the expression of Ki67 and thus decrease tumour proliferation. It can also increase the phosphorylation of P53 in the early phases of breast cancer and induce cell apoptosis, thus showing promising effects in breast cancer [61]. Women taking propranolol years before breast cancer diagnosis showed a reduced rate of metastatic cancer and a lower mortality rate compared to women who were not on treatment with propranolol [58].

Drugs that inhibit the renin-angiotensin system (RAS), such as angiotensin-converting enzyme inhibitors (ACEIs) and angiotensin-receptor blockers (ARBs), are some of the most widely used antihypertensive medications. Lately, there has been strong evidence that RAS is involved in inflammation, cell proliferation via active peptide angiotensin-II signaling, VEGF-mediated angiogenesis in malignancy and metastasis. Hence, drugs that inhibit RAS may slow down tumour growth, especially when used for a long time [62]. ACEIs may be correlated with a lower risk of prostate cancer, pancreatic cancer, colorectal, esophageal and gastric cancer [63,64,65,66]. However, there are data in the literature that try to demonstrate that the administration of ACEIs can increase the risk of lung cancer [67]. This can be explained by the existence of bradykinin and many related receptors in lung cancer tissue, thus releasing VEGF that stimulates angiogenesis. The risk of lung cancer is directly proportional with the dose and the duration of ACEIs use. One study showed that hypertensive patients taking ACEIs versus ARBs had a greater risk of pulmonary cancer [68].

Some experimental studies suggested that metformin, the most commonly prescribed drug for type II diabetes mellitus, displays antineoplastic effects, thus leading to a lower incidence of certain types of cancers in diabetic patients (liver, colon, stomach, breast cancer) [69,70]. Metformin may reduce the risk of cancer by 30 to 50% when compared to insulin or sulfonylureas [28].

The effects of CV drugs on various types of cancer are summarized in Table 1. 

### 3.4. Cardiac and Vascular Toxicity Caused by Antineoplastic Therapy

The increased number of cancer survivors is a direct consequence of the development of novel cancer therapies. These patients, though, are at high risk of developing CV disease because of the effect of cancer on the heart and, also, as a result of chemo- and/or radiotherapy. Risk factors for cardiac and vascular toxicity caused by cancer treatment include preexisting CV risk factors, combination of chemo- and radiotherapy, accumulating doses of drugs or left ventricular dysfunction prior to the initiation of therapy. 

Cancer-related cardiac dysfunction can develop many years after the initial treatment, occurring with an incidence of 10% [71]. HF is the most concerning complication of cancer therapy, followed by myocarditis, coronary artery disease, valvular disease, arrhythmias, arterial hypertension and many other CV complications [72]. The development of CV toxicity significantly worsens the prognosis of oncological patients and may require dose-reduction or even discontinuation of chemotherapy. The pathophysiological classification of cardiotoxicity includes type 1, characterized by dose-dependence and irreversible myocardial damage, and type 2, characterized by dose-independence and reversible myocardial damage. Additionally, other mechanisms are involved in the development of cardiac dysfunction, such as inflammation, conduction abnormalities, arterial spasm and development of hypertension. Anthracyclines (e.g., doxorubicin—DOX) have shown well-established efficacy for the treatment of solid tumours (e.g., breast, gastric, ovarian cancer) and hematological malignancies, but they may also lead to irreversible cardiac damage, which is cumulative and dose-dependent. Anthracycline-induced cardiac dysfunction (AICD) is a result of the progressive decline of cardiac function, which may lead to dilated cardiomyopathy. However, a recent study showed that AICD was associated with hypokinetic non-dilated cardiomyopathy rather than typical dilated cardiomyopathy [73]. Women are less prone to develop AICD when compared to men. This may be a consequence of estrogens that are considered cardioprotective, as they enhance myocardial resistance to ischemia by attenuating abnormal oxidative stress and apoptosis [74]. 

Cardiac arrhythmias are also a consequence of antineoplastic drug use. The most common arrhythmias in cancer patients are prolonged QT interval, atrioventricular block, atrial fibrillation and ventricular arrhythmias. Bradycardia is a consequence of treatment with paclitaxel, thalidomide, palazonib and sunitinib, whereas atrial fibrillation is caused by anthracycline, ibrutinib, melphalan and paclitaxel [75,76]. Chemotherapy is also responsible for prolongation of the QT interval, a condition that resolves gradually as the drugs are being metabolized [77]. 

In addition to cardiotoxicity, chemotherapy is also responsible for vascular toxicity resulting in arterial toxicity (atherosclerosis, coronary artery disease) as well as venous toxicity (venous thromboembolism: deep vein thrombosis or pulmonary embolism). Treatment with paclitaxel can lead to coronary spasms and even MI [78]. Cisplatin has a dose-dependent effect and can cause coronary artery disease [79]. A recent study revealed an increased incidence of MI during treatment with 5-fluorouracil, whereas its use is associated with an increase in copeptin and rarely in cardiac troponin I [80]. Vascular complications are common in cancer, pulmonary cancer patients having the highest rate of mortality, whereas colon cancer patients have the highest bleeding risk [81]. Venous thromboembolism is the second cause of mortality in patients with cancer, after disease progression. Pulmonary embolism develops in about 50% of patients with untreated deep vein thrombosis [82]. The factors that predispose patients with cancer to venous thromboembolism are the type of cancer, radiotherapy, central venous catheter chemotherapy and medication side effects [83]. Arterial hypertension may appear as a result of VEGF signals inhibitors that lead to an imbalance between vasodilator and vasoconstrictor factors. The development of arterial hypertension in the early stages of treatment may be considered a predictor of the medication effect [84]. Therapy with platinum (e.g., oxaliplatin) may induce arterial hypertension and also myocardial ischemia [85]. 

The effects of chemo- and radiotherapy on CV disease are resumed in Table 2.

The general approach to prevent CV toxicity caused by chemotherapy includes pre-treatment estimation of patient-specific risk. Baseline risk assessment is recommended to all patients who will receive a cancer treatment with potential cardiotoxicity and includes, among others, cardiac history, CV risk factors, cardiac troponin, and B-type natriuretic peptide (BNP) or its N-terminal fragment (NT-proBNP), electrocardiogram and echocardiography, as well as cancer treatment history [94]. However, research efforts have lately focused on the identification of other biomarkers of cancer-related CV toxicity, which would predict more accurately the development of CV disease and would allow for intervention in the preclinical phase.

## 4. Mediators Connecting Cardiovascular Disease and Cancer

The pathophysiological overlap between cancer and CV disease is expressed at different levels, including inflammation, oxidative stress, neuro-hormonal activation, clonal hematopoiesis and circulating factors. Traditionally, the interest was to find predictors for CV toxicity associated with antineoplastic treatment and to identify these patients in whom chemotherapy would represent a heavy burden. Nowadays, the relationship between cancer and CV diseases is no longer unidirectional. 

### 4.1. Circulating Factors

The association between HF and cancer was supported initially by experimental studies. In one such study, investigators studied whether certain biomarkers secreted by the failing hearts in mice would promote intestinal tumour proliferation. In this model, HF was the result of induced large anterior MI. The presence of HF resulted in a statistically significant increase in intestinal tumours, both for native and for heterotopically transplanted hearts in mice. Data from the experimental study were extrapolated to a human cohort, which included 180 healthy subjects, and also to approximately 100 patients with chronic HF [40]. The authors selected five proteins considered to be involved in the pathophysiological overlap between HF and cancer, of which SerpinA3, also known as α-1-antichymotrypsin, was increased in patients with HF and promoted cell proliferation in colon tumours. SerpinA3 belongs to the protein superfamily of serine protease inhibitors, which are involved in various inflammatory processes. Data from the literature suggest that levels of serpinA3 predict long-term mortality in patients with HF and are also associated with several cancer types, including colorectal, gastric, breast, liver and prostate cancer [95,96]. This association could have therapeutic implications, as treatment with mineralocorticoid receptor antagonists seems to downregulate the expression of serpinA3, but this needs to be further investigated [97]. Furthermore, the potential effects of serpinA3 on endothelial cell function or cardiac myocytes as contributing to HF and proliferation-associated genes (MCM6, FKBP10 and IGFBP2 in glioma) following activation of MAPK/ERK1/2, PI3K/AKT signalling in endometrial carcinoma are of interest and require further studies [95,98,99]. 

Other mediators that lie at the intersection of cancer and CV disease are tumour biomarkers, which have established roles in the early detection of different cancer types and for monitoring tumour progression. Moreover, certain tumour biomarkers, such as CA15-3, CEA and CA125 represent independent markers for CV disease and predict all-cause mortality in such patients, suggesting that there is still much to consider about the strong association between cancer and CV disease. One study showed that CA15-3 was strongly associated with CV mortality and HF, even after multivariable adjustment for common risk factors, whereas the correlation with coronary artery disease and stroke was not statistically significant. CEA was also associated with CV mortality, after adjustment for cofounders [100]. Inflammation has arisen as a viable cause both in CV disease and cancer, because correlation between tumour biomarkers and proinflammatory cytokines, such as TNF-α, IL-6 and IL-10, has been identified [101]. In addition, it was demonstrated that CA125 secretion could be enhanced by the inflammatory cytokines IL-1β, TNF-α and the lipopolysaccharide of Escherichia coli [102]. The proposed mechanism by which systemic inflammation affects CA125 concentrations involves JNK molecular pathways [103]. These data support the idea that CV disease and cancer share common ancillary mechanisms and pathways, which should be addressed in the future in order to ensure precise phenotyping of both diseases.

### 4.2. Inflammation

Chronic inflammation is involved in both CV disease and cancer. Inflammation is an important part of tumourigenesis, as it contributes to cancer initiation, proliferation and metastasis. The initial evidence for an association between inflammatory markers, CV disease and cancer originated from observational studies. 

#### 4.2.1. Cytokines

Elevated levels of C-reactive protein (CRP), interleukin-1 (IL-1) and interleukin-6 (IL-6) are associated with atherosclerosis and its complications, including plaque initiation, progression and rupture. Increased levels of CRP were also strongly associated with CV disease and cancer mortality [104]. Data from observational studies also revealed that increased levels of IL-6 were associated with both coronary heart disease and cancer [105,106]. However, these seem to be only observational associations, as data from genetic studies argues against a causative role of either CRP or IL-6 in the development of CV disease. IL-1 levels are increased in patients with CV disease, particularly chronic or decompensated heart failure, with increasing levels according to disease severity [107]. The most striking evidence for the role of IL-1 in the development of CV disease and cancer came from the CANTOS trial. In this randomized controlled trial, treatment with canakinumab, a monoclonal antibody targeting IL-1β, decreased the primary end-point (nonfatal MI, nonfatal stroke and CV death) by 15%. This drug was also associated with a decreased incidence and mortality of lung cancer, although this was not a primary outcome [108]. This study represented additional proof that inflammation links CV disease and cancer, acting as a central player in both diseases.

In contrast to cytokines, whose increased levels have been associated with both CV disease and cancer, there are some cytokines, such as IL-10, that play a role in the development of cancer, but with protective effects in CV diseases. Studies have shown that IL-10 has cardioprotective properties in diabetic mice with MI via regulation of heme clearance pathway, by reducing the size of infarction and improving cardiac function [109]. Furthermore, IL-10 improved capillary density, reduced apoptosis and decreased inflammation in the border zone of the infarcted hearts [109]. The anti-inflammatory action of IL-10 was reflected by inhibiting the secretion of other proinflammatory cytokines including TNF-α [104], nuclear factor-κB (NF-κB) and mitogen-activated protein kinases (MAPKs) [110]. In addition to its involvement in CV diseases, IL-10 could also serve as a biomarker for prognostic diseases or as a treatment target when taking into consideration the elevated concentration in cancer patients versus the healthy ones. High levels of IL-10 were observed in patients with Hodgkin’s lymphoma, gastric, pancreatic and pulmonary cancer, whereas IL-10 levels in patients with colon and renal cancer did not differ significantly from the controls [111,112,113]. Hence, IL-10 is considered an independent prognostic factor and correlates with poor survival.

Another interesting marker that is found at the intersection of hematologic malignancies and CV disease is clonal hematopoiesis of indeterminate potential (CHIP), defined as the presence of somatic mutations in hematopoietic cells, occurring among older persons and in the absence of other hematologic abnormalities. These somatic mutations, most frequently involving the genes DNMT3A and TET2, are involved in DNA methylation and have an established role in the development of myelodysplastic syndromes and acute myeloid leukemia. Moreover, CHIP emerged as a potent, independent risk factor for atherosclerotic CV disease, by regulating pro-inflammatory pathways in monocytes and macrophages, which are known to play important roles in the development of atherosclerosis. The presence of CHIP resulted in an almost two-fold increase in the risk of developing coronary heart disease, after adjustment for common risk factors. The association between CHIP and MI was even more remarkable, with a four-fold increase in the risk of developing early-onset MI for patients with CHIP. This causal association is supported by the results from a murine model, showing that mice engrafted with TET2 bone marrow had more significant atherosclerotic lesions than control mice [114]. Overall, the effect of promoting atherosclerosis is not explained by the opposing enzymatic activities of DNMT3A and TET2 on DNA methylation, but rather it is a marker of their non-enzymatic activity, which is yet to be further explored [115]. Interesting, the ERBB2 protein, or HER2/neu (transmembrane tyrosine kinase receptor), a protein that in humans is encoded by the ERBB2 gene, is implicated in the pathogenesis of multiple cancer types and intersection between cancer and chronic HF [116]. 

Recent studies have identified new factors called “cachexokines, which could be useful as biomarkers for the diagnosis of cancer-induced cardiac complications and might lead to the identification of new therapeutic targets. Shafer et al. developed a preclinical study and analyzed two types of mouse colon cancer cells that distinguish themselves by their capacity to induce cardiac cachexia [117]. The authors identified a group of seven secreted proteins, collectively called cachexokines (Bridging integrator 1, Syntaxin 7, Multiple inositol-polyphosphate phosphatase 1, Glucosidase alpha acid, Chemokine ligand 2, Adamts like 4 and Ataxin-10), which lead to cardiomyocyte atrophy and aberrant fatty acid metabolism in cardiomyocytes. The simultaneous downregulation of the seven cachexokines on colon cancer cells blocks their capacity to induce cardiac atrophy, whereas the blockage of the single proteins does not influence the cachectic phenotype. The same study showed that ataxin-10 correlates with cachexia, both in mouse models and in pancreatic cancer patients. These findings may pave the way for personalized predictive, diagnostic and therapeutic measures in cancer cachexia.

#### 4.2.2. Immune Cells

Dysregulation of the immune system is involved in both cancer and CV disease. In cancer, an insufficient or inadequate immune response is responsible for tumour development and progression. The role of the immune system in CV disease is also crucial. Within hours following an acute MI, neutrophils gather in the infarcted area, followed by monocytes and macrophages in subsequent days. The role of cardiac macrophages is also prominent in HF with preserved ejection fraction, promoting cardiac remodelling and fibrosis. In a mouse model of breast cancer, it was showed that MI accelerated tumour cell proliferation, resulting in an approximately two-fold increase in tumour volume and weight at 20 days [118]. This proliferative effect was demonstrated by staining for Ki67+ tumour cells before and after inducing MI, resulting in a doubling of Ki67+ cells in the tumour border of mice with MI versus sham procedure. Further on, the authors attempted to discern the mechanisms by which MI promotes tumour proliferation. They found that MI determines an accumulation of CD11b+Ly6Chi monocytes in tumours, which share similar markers with monocytic myeloid derived suppressor cells, suggesting that MI promotes an immunosuppressive intratumoural response [118]. The authors concluded that MI induces complex epigenetic reprogramming of myeloid cells in hematopoietic reservoirs, resulting in an immunosuppressive phenotype in breast cancer that promotes tumour proliferation [118]. These preliminary results confirmed that dysregulation of the immune system, as a consequence of an acute CV event, influences tumour progression in an animal model. As stated previously, observational studies found an association between MI and cancer development in humans, but these data need further confirmation through rigorous, specifically designed trials. 

### 4.3. Neuro-Hormonal Activation

Among the complex pathophysiological pathways of HF, renin–angiotensin–aldosterone system (RAAS) plays a central role, initially as a compensatory mechanism, and as the disease progresses, when it becomes maladaptive, leading to cardiac remodelling and sympathetic activation. The main mediator of RAAS, angiotensin II (AngII), which acts by binding to either type 1 Ang II receptor (AT1R) or type 2 Ang II receptor (AT2R), has been associated with detrimental effects, generally promoting cardiac hypertrophy, vasoconstriction and inflammation, or with beneficial effects, including anti-inflammatory and cardio-protective effects, respectively. Similarly, RAAS seems to be involved in cancer pathogenesis, with AngII/AT1R axis promoting tumour cell proliferation, inhibiting apoptosis and creating a pro-inflammatory and immunosuppressive tumour microenvironment. The involvement of RAAS in cancer pathogenesis would naturally raise the therapeutic question whether RAAS blockade with either ACEIs or ARBs would improve cancer survival. The first meta-analysis that investigated the effects of an RAAS blockade on cancer recurrence and survival demonstrated a statistically significant risk reduction of 40% and 25%, with the use of ACEI or ARB [119]. Another meta-analysis indicated a significant improvement in overall survival and cancer-specific survival with the use of RAAS blockers in patients with digestive system malignancies [120]. However, the involvement of RAAS in cancer pathogenesis seems to vary according to cancer type and several studies did not find a beneficial effect of treatment with RAAS inhibitors on cancer survival [119]. There is still much to be understood about the implications of RAAS in the pathophysiology of cancer. 

## 5. Role of Extracellular Vesicles in Cross Talk between CV Disease and Cancer

In recent years, studies have shown that biological fluids display different types of vesicles released by the vast majority of cells, tissues and organs. These vesicles, with the classic general name of extracellular vesicles (EVs), have local or remote effects, participating in various physiological and pathological processes. Interestingly, they appear to maintain bidirectional communication between the heart and other organs and play a particularly important role in cardiac physiology and pathophysiology [121].

### 5.1. What Do We Know about Extracellular Vesicles?

The term EVs refers to all types of nanovesicles released into the extracellular space and subsequently into various biofluids including blood by apoptotic or activated cells, both in physiological and pathological conditions. Depending on the mechanism through which these vesicles are released, but also on their size, they have been classified into exosomes and microvesicles (also called microparticles or ectosomes) [122,123]. Additionally, some studies include apoptotic bodies (also called apoptosomes) in the large family of EVs [124]. Recent studies have adopted a new nomenclature for EVs, namely small and large vesicles [125]. The classification of EVs is constantly changing, but today it is accepted that EVs are represented by exosomes, microvesicles and possibly apoptotic bodies, based on their biogenesis, and small and large EVs based on their size. Current investigations consider several critical aspects of EVs, namely choosing the optimal separation method, finding the best storage conditions and establishing optimal characterization protocols and therapeutic strategies and dosages. Separation of large and small EV-enriched subpopulations from cardiac progenitor cells was efficiently achieved with two commonly purification methods: ultracentrifugation and size-exclusion chromatography [126]. As for their characterization, the small EVs (exosomes and microvesicles, with a diameter of ~100 nm), that sediment at 100,000× *g*, contain ALIX and CD63, and large EVs (apoptotic bodies, with a diameter >1000 nm), that sediment at 10,000× *g*, are abundant in calnexin [126]. In addition to these proteins specific to each subpopulation of EVs, they also contain a great variety of proteins, lipids and nucleic acids that reflect the cytosolic content of the source cell [127,128]. When comparatively investigating their functionality, no differences were found between small and large EVs [126]. The correct classification and characterization of EVs are important elements in completing the following steps, namely establishing their ability as biomarkers and therapeutic tools for various pathologies, including cancer and CV disease.

### 5.2. Extracellular Vesicles as Communication and Transport Entities

EVs are nanosized vesicles delimited by a lipid bilayer that contain various biologically active molecules (enzymes, proteins, transcription factors, lipids, and nucleic acids: DNA, mRNA, microRNA (miRNA), long non-coding RNA, circular RNA), and have the ability to reach all organs, tissues and cells of the body after their secretion into biological fluids. Almost any type of cells (endothelial cells, lymphocytes, T cells, macrophages, renal cells, cancer cells and stem cells) can release EVs in physiological state, and a number of these submicron vesicular membrane structures is increased during pathological processes [129]. Therefore, EVs can provide diagnostic and prognostic value in a variety of pathologies such as: chronic venous insufficiency, atherosclerosis and CV disease, ischemic stroke, diabetes, COVID-19, but also cancer [130,131,132,133,134,135,136,137,138,139]. In addition to their role as disease biomarkers or communication entities in different physiological ant pathological situations, EVs have long been studied for their influence as therapeutic tools, especially in atherosclerosis and CV disease [140,141,142,143]. Their potential as drug delivery systems has been recently noticed and disseminated in different medical applications: cardiac regeneration, cancer treatment and, also, in nuclear medicine as radionuclide carriers [144]. An opinion paper on EVs as theranostic agents discussed the benefits of radio-labeled EVs in diagnostic and interventional medicine [145]. The role of EVs in the development or regression of atherosclerotic lesions has been widely discussed, and the link between thrombosis and inflammation has been emphasized [146]. EVs from subcutaneous adipose tissue stem cells and bone marrow mesenchymal stem cells, loaded or not with Smad2/3-siRNA, ameliorated vascular dysfunction and reduced atherosclerosis-associated inflammation in an experimental animal model of atherosclerosis [142]. In addition, the same types of EVs significantly decreased specific hypertrophic markers and molecules involved in the inflammatory process associated with cardiac hypertrophy in an in vitro model of human-induced pluripotent stem cell-derived cardiomyocytes [143]. These effects were partially related to the miRNAs that EVs contain [142,143]. These findings suggest that cell-free therapy based on stem cell-derived EVs may provide new alternatives for therapeutic strategies targeting CV disease.

At present, there are quite a few studies that have researched and discussed the potential connection between CV disease and cancer. 

### 5.3. Extracellular Vesicles as Nano Mediators in Cancer and Associated Cardiovascular Disease

EVs are considered potential mediators through which tumour cells communicate with each other and influence their microdomain promoting tumour progression and dissemination, by altering the immune response that subsequently generates cell proliferation, angiogenesis, matrix remodelling and finally metastasis [147,148]. EV contribution to tumour angiogenesis, neovascularization and hypoxia-dependent inter-tumour communication during cancer progression was demonstrated on metastatic brain tumour glioblastoma [149].

Generally, EVs were investigated as biomarkers for initial diagnosis, patient follow up, and, also, for evaluation of tumour response to therapy [150,151,152].

It is now well known that all cells, especially tumour cells, secrete heterogeneous EV subpopulations that vary in size, content, and function. EVs contain on their surface receptors specific to the tumour cells from which they come and specific to the type of cancer. Furthermore, their intravesical content depends on the type and stage of cancer. For instance, during hypoxia, EVs released from cancer cells are enriched in angiogenic factors, such as VEGF and hypoxia-inducible factor 1-alpha (HIF-1α) that promote angiogenesis and later tumour metastasis [153]. In ovarian cancer, EVs carrying VEGF contribute to angiogenesis and metastasis by crosstalk between cancer and endothelial cells [154]. In addition, glioblastoma-specific small EVs are demonstrated to contain the epidermal growth factor receptor variant III (EGFRvIII), the oncogenic mutant variant of EGFR frequently detected in glioblastoma tumours, which stimulates VEGF production and promotes angiogenesis [155]. Interestingly, the EGFRvIII mRNA levels were comparable in large and small EVs isolated from U87^EGFRvIII^ cells, whereas large EVs had higher levels of EGFR protein (EGFRwt + EGFRvIII) compared to small EVs [125]. Thus, EGFRvIII mRNA and protein status can provide glioblastoma-specific liquid biopsy-based monitoring that can help not only diagnose but also evaluate tumour progression [155]. The role of EVs and their miRNA content as diagnostic, prognostic and disease monitoring biomarkers and as nanocarriers for gene therapy in glioblastoma progression and resistance to therapy have been widely debated in a recently published paper [139].

In breast cancer, EVs encompass epidermal growth factor-like repeats and discoidin I-like domains 3 (EDIL3), a glycoprotein that activates the integrin-FAK signaling cascade and plays a crucial role in cancer development [156,157,158]. EDIL3 is also known as a novel regulator of epithelial-mesenchymal transition in clear cell renal cell carcinoma [159]. EDIL3 on EVs also contribute to the enhancement of cell invasion and acceleration of lung metastasis in vivo [156]. Consequently, it was suggested that EDIL3 might be employed as a therapeutic target that limits disease progression.

There are a number of studies that have shown the primary role of EVs in the various stages of cancer and especially in tumour metastases. In addition, the role of EVs in CV pathology is well known and has been studied for a long time.

Very little data concern the involvement of EVs in cardio-oncological pathology, although intuitively, it is becoming increasingly clear that EVs could have a substantial effect.

**Figure 2:**
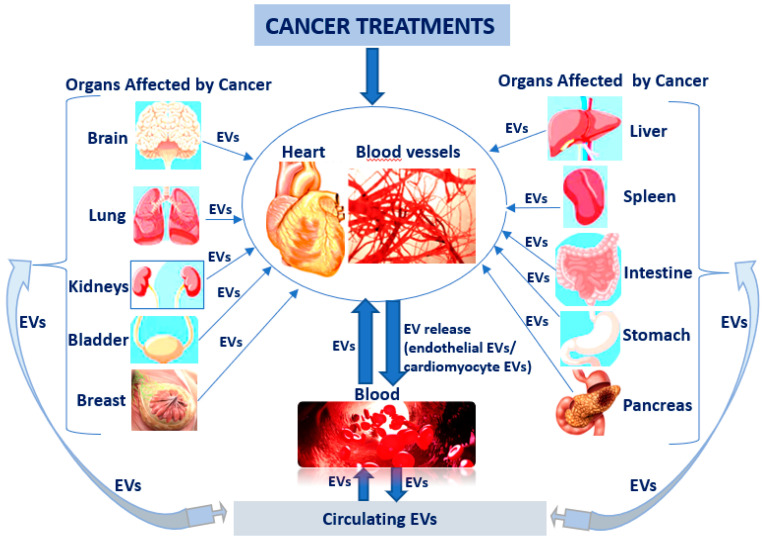
EVs as key biomarkers in cardio-oncological pathology: cancer therapy affects the heart and blood vessels and generates the release of endothelial and cardiomyocyte EVs into circulation, which can subsequently disturb the other organs; EVs released into circulation from apoptotic, activated or tumour cells can reach any other organ, including heart and blood vessels.

It is already known that hormone therapies used to treat breast and prostate cancers can raise the risk for a heart attack and stroke. In this case, patient follow-up is very important to identify the first cardiac changes manifested by a reduction of left ventricular ejection fraction, but finding early biomarkers could also have a decisive role in the early treatment of CV diseases. EVs could be one of the key biomarkers, especially since it is known that once released into circulation from apoptotic or activated cells (comprising tumoural cells) can potentially reach any organ, including heart and blood vessels. Furthermore, EVs can be mediators in reverse communication between heart and skeletal muscle, kidneys, bone marrow, lungs, liver, adipose tissue, and brain (Figure 2). 

In this research direction, the potential of exosomes (small EVs) as diagnostic biomarkers and therapeutic carriers for DOX-induced cardiotoxicity was discussed by Tian et al. in 2021 [160]. DOX, known as a cytotoxic anthracycline antibiotic used to treat several different types of cancer, kills not only tumour cells through circulation but also damages cardiomyocytes at the same time and induces senescence and various death forms of cardiac cells, such as apoptosis, necroptosis, autophagy, pyroptosis and ferroptosis [160]. 

#### 5.3.1. EVs as Diagnosis Biomarkers in Cardio-Oncology

Regarding EVs’ biomarker capacity, in a DOX-induced cardiac injury mouse model, it was shown that cardiomyocytes release EVs that contain protein biomarkers (4-hydroxynonenal (HNE) and brain-type glycogen phosphorylase (PYGB)) of early cardiac injury, prior to cardiac troponin [161]. In addition, these EVs can be used to predict the antioxidant capacity against the chemotherapy of individual patients and to determine the dose of chemotherapy that will avoid normal tissue injury [161]. Thus, mice which develop antioxidant capacity after treatment with manganese superoxide dismutase (MnSOD) or cationic manganese (III) ortho N-substituted pyridylporphyrins (MnP)-pretreated mice have low levels of circulating EVs following DOX treatment. Furthermore, administration of dexrazoxane (DRZ), a clinically approved cardioprotective drug that has antioxidant effects, diminished EV release from heart tissue. However, the effect of DRZ on EV release was weaker than that of MnP, which might be explained by the high preference for the mitochondria of MnP.

Of note, miRNAs contained by EVs could have potential as biomarkers for early detection of DOX-induced cardiotoxicity [162]. Specifically, EVs-miR-502 found to be upregulated after single-DOX chemotherapy in dog model of sarcoma may be a damage marker for DOX-induced cardiotoxicity [162]. In addition, upregulation of miR-502 and downregulation of miR-107 and miR-146a were detected in EVs before any significant changes were seen in other established biomarkers, including cardiac troponin I (cTnI) and echocardiographic parameters (left ventricular ejection fraction and left ventricular internal dimension in diastole). It is worth mentioning that exosomal miRNAs were more persistent compared to cardiac troponin I (cTnI) in serum. 

#### 5.3.2. EVs as Therapeutic Delivery Tools in Cardio-Oncology

On the other hand, EVs themselves, because of their intravesical content could be important messengers for the regulation of various disturbed biological processes or ideal carriers for chemotherapeutic drugs to alleviate cardiotoxicity. Thus, large EVs from mesenchymal stem cells (MSCs) mitigated the negative effects induced by 1 μmol/L DOX for 24 h on induced pluripotent stem cell (iPSC)-derived cardiomyocytes [163]. These large EVs, found to be enriched in mitochondria, improved contractility, reactive oxygen species production, ATP production and mitochondrial biogenesis in cardiomyocytes [163]. Interestingly, inhibiting mitochondrial function with 1-methyl-4-phenylpyridinium attenuated the beneficial effects of EVs on cardiomyocyte injury induced by DOX treatment [163]. This exciting study was based on the SENECA Trial, a Phase 1 National Heart, Lung and Blood Institute-sponsored study that evaluated the role of MSCs in treating anthracycline-induced cardiomyopathy but without emphasizing the biological mechanisms involved. 

Exosomes^Hypoxia^ isolated from human adipose-derived MSCs pretreated with hypoxia had a better cardioprotective effect then exosomes from untreated MSCs on human induced pluripotent stem cell (iPSC)-derived cardiomyocytes exposed to 0.5 μM DOX for 24 h [164]. LncRNA-MALAT1/miR-92a-3p/ATG4a partially mediated the cardioprotective roles of exosome^Hypoxia^ in DOX-induced cardiac damage [164]. EVs collected from human iPSC-derived CV progenitor cells increased ATP levels and enhanced both mitochondrial respiration and anaerobic on iPSC-derived cardiomyocytes treated with 0.6 μM DOX (0.2 μM DOX every 48 h) [165]. The positive effects of intravenously delivered EVs on cardiac function have been demonstrated in male BALB/c mice with chemotherapy-induced cardiomyopathy (mice subjected to three weekly injections of DOX: total cumulated dose was of 12 mg/kg body weight) [165]. 

In addition, the intravenous administration of cardiac progenitor cell-derived exosomes protected neonatal rats against DOX/trastuzumab (TRZ)-induced cardiac toxicity through repressing inflammatory responses (attenuating macrophage infiltrates and iNOS expression), decreasing myocardial fibrosis and restoring cardiac function [166]. Particularly, exosomal miR-146-5p mediated some of the cardioprotective effects of exosomes (used at concentration of 3.5 × 10^6^/cm^2^ for 1 h) by suppressing oxidative stress and target genes Traf6, Smad4, Irak1, Nox4 and Mpo in the neonatal rat ventricular myocytes exposed to 1 µM DOX for 3 h and 1 µM TRZ for a further 3 h [166].

Interestingly, exosomes derived from embryonic stem cells inhibited pyroptosis (cell death characterized by the release of vast pro-inflammatory factors) of H9c2 cardiomyoblasts induced by 2 µM DOX for 24 h. Expressions of TLR4, NLRP3, pyroptotic markers (caspase-1, IL-1ß, caspase-11, and gasdermin-D) and proinflammatory cytokines (TNF-α and IL-6) in H9c2 cells were decreased after 24 h of incubation with 10 µg exosomes [167]. The potential beneficial effects of embryonic stem cell-derived exosomes were also considered on DOX-induced pyroptosis and cardiac remodeling in C57BL/6J mice by three intraperitoneal (i.p) injections (4 mg/kg body weight), administrated on alternative days in a week time span (Monday, Wednesday, and Friday) for a cumulative dose of 12 mg/kg. Exosomes (400 µL/injection with 50 µg concentration of exosomes) injected on alternative days between DOX treatments (Tuesday, Thursday, and Saturday) decreased expressions of inflammasome markers (TLR4 and NLRP3), pyroptotic markers (caspase-1, IL1-β, and IL-18), cell signaling proteins (MyD88, p-P38, and p-JNK), pro-inflammatory M1 macrophages and TNF-α cytokine [168]. Moreover, exosomes increased M2 macrophages and the anti-inflammatory cytokine IL-10 inhibited cytoplasmic vacuolization, myofibril loss and hypertrophy, and improved heart function [168]. 

In addition to their abilities as important massagers, owing to their intravesicular content, EVs can also be carriers for drugs or other biological compounds. Relatively recently it has been shown that tumour cell-derived exosomes are home to their cells of origin and can be used as Trojan horses to deliver cancer drugs [169].

Specifically, exosomes from two cancer cell lines, HT1080 or Hela, engineered to carry DOX, systemically injected in nude mice bearing a subcutaneous HT1080 tumour for 15 days, fused preferentially with their parent cancer cells and eradicated tumour tissues more effectively than DOX alone (5 mgDOX/Kg of body weight) [169]. Similarly, in a mouse model of breast and ovarian cancer, it was proven that exosomes increased the therapeutic index of DOX without enhancing the cardiotoxicity of DOX. Thus, exosomes have become common delivery vehicles of DOX to target various cancers and receive better therapeutic effects than free DOX [170]. In conclusion, these exciting results indicated that exosomes derived from cancer patients might be a good weapon in the fight against cancer. This idea must be viewed with caution and studied more widely because of the metastatic role of cancer-derived exosomes.

However, rapid uptake of exosomes by the mononuclear phagocyte system (MPS) remains an obstacle for drug delivery into other targeted organs, including the heart. To solve this problem, Wan et al., hypothesized in 2020 that prior blocking of uptake of exosomes by the MPS would improve their delivery to the targeted organs [171]. They found that clathrin heavy chain (Cltc) plays a significant role in mediating endocytosis of the exosomes in the liver and spleen, and blocking it caused therapeutic exosomes (based miR-21a delivery) to be more efficiently distributed to targeted organs, such as the heart, and produced a much better therapeutic effect on cardiac function in the DOX-induced cardiotoxicity mouse model [171].

Furthermore, the immunogenicity and toxicity generated by EVs might complicate their use as transport vehicles. To reduce these negative effects, the mouse immature dendritic cells (DCs) were used for exosome production in a study conducted by Tian et al. in 2014 [172]. They used these exosomes engineered to express a well-characterized exosomal membrane protein (Lamp2b) fused to αv integrin-specific iRGD peptide (CRGDKGPDC) to deliver DOX to tumour tissue in BALB/c nude mice transplanted to human breast cancer cells (MDA-MB-231). Consequently, tumour growth was inhibited without overt toxicity [172]. 

In the treatment of cancer, the problem of adverse effects, which includes the occurrence of CV diseases, remains unsolved. In this sense, a recent study tried to propose another innovative treatment for cancer based on stimulation of cancer-specific T cells instead of the high-dose DOX treatment that generates cardiotoxicity. Thus, Phung et al. used DC-derived exosomes engineered with anti-CTLA-4 antibody anchored in their lipids as an anti-cancer strategy. In this way, they induced an increase in the response of T cells to the presence of cancer cells [173].

Table 3 summarizes some aspects regarding relationship between EVs and cardio-oncological pathology/therapy.

Recently, the impact of breast cancer therapies on the CV system with a focus on the endothelium and the problem of detecting the early asymptomatic stages of cancer therapy-related cardiac dysfunction by cardiac imaging alone has been highlighted. Unfortunately, the initiating mechanisms of CV changes in patients suffering from cancer are incompletely understood, but the status of circulating biomarkers has emerged as a possible explanation. It was pointed out that the investigation of biomarkers, including inflammatory markers, cardiac biomarkers, miRNAs and endothelial cell-derived EVs may uncover mechanisms of injury, detect early stages of CV damage and elucidate novel therapeutic approaches for cancer therapy-related cardiac dysfunction [174]. 

There are very few studies that show the diagnostic or prognostic role of these biomarkers for the occurrence of cardiomyopathy after chemotherapy, but the addition of other specific biomarkers could help. Some clinical studies highlighted that troponins can be used to assess the cardiotoxicity induced by chemotherapy, specially trastuzumab and several tyrosine kinase inhibitors [175,176,177]. Thus, the quantification of plasma troponin I and T concentrations, which are markers of myocardial cell damage and B type natriuretic peptides, which are cardiac neurohormones secreted specifically by the ventricles in response to increased wall stress, can also be used as indicators of cardiotoxicity [175]. The role of the main two serum biomarkers troponin and natriuretic peptides in cancer patients receiving cardiotoxic cancer therapies (anthracycline chemotherapy, trastuzumab, HER2-targeted therapies, vascular endothelial growth factor inhibitors, proteasome inhibitors, immune checkpoint inhibitors, cyclophosphamide and radiotherapy) was discussed extensively by a Cardio-Oncology Study Group of the Heart Failure Association and the Cardio-Oncology Council of the European Society of Cardiology [178].

Interestingly, positive associations were found between CV markers (high-sensitivity cardiac troponin T, NT-proBNP, myeloperoxidase, placental growth factor and growth differentiation factor 15), cardiac dysfunction and breast cancer therapy (particularly in sequential anthracycline and trastuzumab therapy) [179].

However, liquid biopsy for some relevant circulating biomarkers, including EVs, remains a promising non-invasive method for diagnosis and monitoring CV risk for cancer patients in the near future.

## 6. Conclusions and Future Directions

It is becoming more and more clear that patients suffering from cancer end up developing associated heart diseases over time as a result of the specific therapy administered. Because of the alarming increase in the number of patients with cardio-oncological diseases, clinicians and researchers alike are concerned with optimizing clinical results based especially on CV imaging, and finding relevant biomarkers for the early detection of cardiac dysfunction generated by cancer therapy. With reference to CV imaging, echocardiographic assessment of the left ventricular ejection fraction is limited in the sensitive detection of early, subclinical changes in cardiac function. As a result, the recently appearing large cardio-oncological research groups started to develop platforms and different algorithms to investigate novel mechanisms, biomarkers and therapies to improve CV outcomes in cancer patients. 

Circulating biomarkers, including EVs (lipid-covered particles), could serve as a measure for identifying early cardiac changes caused by cancer therapy. A series of questions regarding EVs remain unsolved for the time being, one of them being related to the origin of tumour-specific EVs and their distinction from the rest of EVs originating from other activated but non-malignant cells.

It is increasingly clear that the investigation of EV-based biomarkers in cross talk between CV disease and cancer is in the beginning stages, but it is becoming a general challenge common to clinicians and researchers. The potential of proteins, miRNAs and other nucleic acids contained in EVs remains to be investigated and to be used later as biomarkers for cardiac toxicity.

In addition to their use as diagnostic biomarkers for early detection of cardiac disorders, EVs can be used as therapeutic vehicles for drugs or regulatory molecules (nucleic acids and proteins) to modulate chemo-resistance of cancer cells and decrease the cardiotoxicity induced by DOX. Furthermore, EVs can be engineered to stimulate immune cells to kill cancer cells, thus decreasing the dosage of DOX and attenuating cardiotoxicity. Because of the many benefits of EVs in cancer therapy and the cardiotoxicity generated by cancer-specific treatment, exploring EV-based therapies in cardio-oncological diseases remains an opportunity for the future.

The ultimate goal of multidisciplinary cardio-oncology teams will be to ensure that cancer patients receive appropriate anti-cancer therapy that minimizes any other CV complication, but also to receive specific treatment for associated heart disease assessed before, during and after cancer therapy.

## Figures and Tables

**Table 1:** Effects of CV medication on different types of cancer.

CV Medication	Effects on Different Types of Cancer	References
Aspirin	- ↓ the risk of colorectal cancer	[47]
Thiazide diuretics	- ↑ the risk of skin cancer - ↑ the risk of breast cancer/protective role against breast cancer (further studies are needed)	[51,52,53]
Calcium channel blockers	- ↑ the risk of lung and prostate cancer in men and breast cancer in women	[55,56]
Beta-blockers	- ↓ the incidence of hepatocellular carcinoma in patients with cirrhosis (non-selective beta-blockers) - ↓ the risk of pancreatic cancer (both selective and non-selective beta-blockers) - ↓ the risk of pancreatic cancer and shows promising positive effects on breast cancer (propranolol)	[59,61]
Angiotensin-converting enzyme inhibitors	- ↓ the risk of prostate cancer, pancreatic cancer, esophageal, gastric, and colorectal cancer - ↑ the risk of lung cancer	[63,64,65,66,67]
Metformin	- ↓ the incidence of liver, colon, stomach, and breast cancer	[70]

↑ means increase; ↓ means decrease.

**Table 2:** Effects of chemo- and radiotherapy on CV disease.

Chemotherapy/Radiotherapy	Effects on CV Disease	References
Anthracyclines	- heart failure, myocarditis, cardiac arrhythmias - hypokinetic non-dilated cardiomyopathy or dilated cardiomyopathy	[73,86]
Antimetabolites (e.g., 5-fluorouracil)	- myocardial ischemia, cardiac arrhythmias	[80]
Antimicrotubular agents (e.g., paclitaxel)	- cardiac arrhythmias, prolongation of QT interval, atrioventricular block - coronary spasms, myocardial infarction	[78]
Platinum (e.g., oxaliplatin)	- arterial hypertension, myocardial ischemia, cardiac arrhythmias	[87,88]
Target human epidermal growth factor receptor 2-positive breast cancer (e.g., trastuzumab)	- left ventricular dysfunction, heart failure	[89]
Tyrosine kinase inhibitors	- pulmonary hypertension, prolongation of QT interval, myocardial infarction, stroke, peripheral vascular thromboembolic events	[90]
Immunomodulatory agents (e.g., lenalidomide)	- venous or arterial thromboembolic events	[91]
Immune checkpoint inhibitors (e.g., nivolumab)	- myocarditis	[92]
Radiotherapy	- pericardial calcification, pericarditis, myocarditis, myocardial ischemia, cardiac arrhythmias - mitral, tricuspid, aortic valvulopathy	[93]

**Table 3:** EVs as biomarkers and therapeutic carriers in cardio-oncological experimental models.

Cancer Treatments	Experimental Models	Biological Effects of EVs	References
**EVs as Biomarkers**			
1 μmol/L DOX for 24 h	DOX-induced cardiac injury mouse model	- increased release of cardiomyocyte EVs could be a biomarker for DOX-induced cardiac injury - cardiomyocyte EVs positive for HNE and PYGB have pro-oxidant capacities	[161]
5 doses of DOX (1 mg/kg body weight each dose) given intravenously 2 to 3 weeks	dogs with sarcoma receiving DOX treatment	upregulated EVs-miR-502 could be a biomarker for DOX-induced cardiotoxicity	[162]
1 μmol/L DOX for 24 h	iPSC-derived cardiomyocytes injured with DOX	large EVs from MSCs mitigate negative DOX effects	[163]
**EVs as Therapeutic Carriers**			
0.5 μM DOX for 24 h	in vitro model of cardiac injury: human iPSC–derived cardiomyocytes exposed to DOX	exosomes derived from MSCs pretreated with hypoxia have a cardioprotective effect by modulating lncRNA-MALAT1/miR-92a-3p/ATG4a expressions	[164]
0.6 μM DOX for 48 h	iPSC-derived cardiomyocytes treated with DOX	EVs collected from human iPSC-derived CV progenitor cells increase ATP levels and enhance both mitochondrial respiration and anaerobic	[165]
12 mg/kg DOX for 3 weeks	BALB/c mice subjected to injections of DOX	EVs from iPSC-derived CV progenitor cells recover cardiac function	[165]
six doses of DOX (Days 1–11, cumulative dose = 15 mg/kg), followed by six doses of TRZ (Days 19–28, cumulative dose = 20 mg/Kg)	neonatal rats with DOX/TRZ-induced cardiac toxicity	cardiac progenitor cell-derived exosomes have therapeutic potential: can repress inflammatory responses, decrease myocardial fibrosis and restore cardiac function	[166]
1 µM DOX for 3 h and 1 µM TRZ for further 3 h	neonatal rat ventricular myocytes exposed to DOX/TRZ	exosomal miR-146-5p suppresses oxidative stress and targets genes Traf6, Smad4, Irak1, Nox4 and Mpo, and is partially involved in cardioprotection	[166]
2 µM DOX for 24 h	in vitro cell culture model of DOX-induced pyroptosis in H9c2 cardiomyoblasts	exosomes (10 µg for 24 h) derived from embryonic stem cells inhibite pyroptosis: diminished expressions of TLR4, NLRP3, pyroptotic markers (caspase-1, IL-1ß, caspase-11, gasdermin-D) and proinflammatory cytokines (TNF-α, IL-6)	[167]
12 mg/kg DOX for 1 week	C57BL/6J mice injected with DOX to induce cardiomyopathy	exosomes (150 µg for 1 week) derived from embryonic stem cells: - ameliorate pyroptosis and cardiac remodeling: - decrease expressions of inflammasome markers (TLR4, NLRP3), pyroptotic markers (caspase-1, IL1-β, IL-18), cell signaling proteins (MyD88, p-P38, p-JNK), pro-inflammatory M1 macrophages and TNF-α cytokine - increase M2 macrophages and anti-inflammatory cytokine, IL-10 - inhibite the cytoplasmic vacuolization, myofibril loss, hypertrophy - improve heart function	[168]
3 × 10^11^ tumour cell-derived exosomes labelled with 5 µg/ml DOX for 15 days Or 5 mg/Kg of body weight DOX for 15 days	nude mice bearing a subcutaneous HT1080 tumour as a model	tumour cell-derived exosomes engineered to carry DOX fuse preferentially with their parent cancer cells and eradicate tumour tissues more effectively than DOX alone	[169]
5 mg/kg/week DOX for 4 consecutive weeks	DOX-induced cardiotoxicity mouse model	injection of 200 µg exosomes loaded with siRNA Cltc 3 days before DOX treatment, followed by injection with 200 µg therapeutic exosomes (loaded with miR-21a) repeated every week for 4 weeks of DOX treatment significantly protect myocardium from apoptosis	[171]
100 µg DC-derived exosomes engineered to express Lamp2b fused to CRGDKGPDC and labelled with 50 µg DOX	BALB/c nude mice transplanted with human breast cancer cells (MDA-MB-231, 2.0 × 10^6^ cells in 50 mL PBS)	intravenously injected DC-derived exosomes engineered to express Lamp2b fused to CRGDKGPDC delivered DOX specifically to tumour tissues, leading to inhibition of tumour growth without overt toxicity	[172]

## Data Availability

The data presented in this study are available on request from the corresponding author.
